# Diet, digestion and energy intake in captive common marmosets (*Callithrix jacchus*): research and management implications

**DOI:** 10.1038/s41598-019-48643-x

**Published:** 2019-08-20

**Authors:** Michael L. Power, Jessica Adams, Kirsten Solonika, Ricki J. Colman, Corinna Ross, Suzette D. Tardif

**Affiliations:** 1 Conservation Ecology Center, Smithsonian Conservation Biology Institute, Washington DC, USA; 2 Southwest National Primate Research Center, Texas Biomedical Research Institute, San Antonio, TX USA; 3 Wisconsin National Primate Research Center, Madison, WI USA; 4 Department of Cell & Regenerative Biology, School of Medicine and Public Health, University of Wisconsin, Madison, WI USA; 5 Texas A&M University San Antonio, San Antonio, TX USA

**Keywords:** Physiology, Zoology

## Abstract

Common marmosets (*Callithrix jacchus*) are susceptible to intestinal inflammation which leads to chronic diarrhea, weight loss, and vitamin D deficiency. We examined food intake and digestion in three mixed-sex groups of adult marmosets maintained on three commercial base diets. Animals underwent two consecutive 4-day digestion trials. Body mass stayed constant. Feces and diet were assayed for Mn, fat, and gross energy (GE). Apparent digestibility of dry matter (ADDM) was calculated by the total collection method and from dietary and fecal Mn; the methods produced correlated results (r = 0.658, p < 0.001). Apparent digestibility of energy (ADE) was calculated from ADDM and the GE of feces and diet; apparent digestibility of fat (ADfat) was calculated from ADDM and fecal fat. ADDM and ADE varied by diet (p < 0.001). We found poor digesters on all three diets. The concentration of fecal fat was inversely related to ADE (r = −0.729, p < 0.001). High fecal fat (>10%) was associated with ADfat of zero, consistent with lipid malabsorption. Mean digestible energy intake (DEI) was equal to 1.5 the estimated metabolic rate, but varied widely between individuals. The diet with the fewest animals with high fecal fat had the highest mean DEI and most animals above 450 g, suggesting it may be obesogenic.

## Introduction

The marmoset is an established animal model used in multiple biomedical research areas, including neuroscience, infectious disease such as hemorrhagic fevers, behavioral research, obesity, and reproductive biology^1^. The primary advantages of this New World monkey as a model are related to its small size (300–450 g), short life span and high fecundity^2^. However, use of the marmoset as a model in human health studies are hampered by an incomplete understanding of their nutritional requirements and lack of standardized dietary husbandry. The literature regarding nutritional requirements in marmosets is sparse, with most published reports providing information on a limited number of specific diet components in relatively small numbers of animals^3^. Dietary regimes are more influenced by anecdotal experience and animal food preferences than by solid evidence, which has led to a wide variety of dietary regimes among institutions housing marmosets ranging from cafeteria style kitchen made diets, to commercial purified irradiated diet with no fresh foods^3–5^. This variability in dietary husbandry may result in high variation in nutrient intakes between individuals, both within and between colonies, which can have metabolic and physiological consequences that can contribute to unexplained variation in experimental outcomes^3^. The different dietary husbandry regimes also may contribute to some of the common clinical diseases observed in this species in captivity^3^.

Captive marmosets display several debilitating diseases that have potential links to nutrition and dietary husbandry including intestinal inflammation, metabolic bone disease, and obesity with associated insulin resistance^6^. Obesity prevalence in marmoset colonies has been increasing^5–8^ and may indicate an overestimation of energy requirements for this species in captivity or possibly diets that are highly palatable and lead to overeating^3,9^. In contrast, susceptibility to inflammatory bowel disease (IBD), in some form, has been implicated in poor weight gain in young marmosets^10^ and weight loss in older animals, with associated higher morbidity and mortality^6,10^. This susceptibility to IBD is a major limitation in developing the common marmoset as a laboratory animal for biomedical research. Initially, the disease was described as causing predominately colitis; however, recent data indicates that the disease can become much more severe in the small intestine and has been termed chronic lymphocytic enteritis or CLE^6,11,12^. Regardless of presentation, the cause has remained elusive, although treatment to remove specific pathogens from primate colonies has met with some success at decreasing the incidence. The disease is characterized by chronic diarrhea and progressive weight loss that leads to decreases in serum albumin^12,13^. Additional secondary findings include osteoporosis, anemia, and ill-thrift. In addition to issues associated with identifiable IBD, subclinical gut inflammation may alter effective captive management and research use by resulting in altered digestive efficiency, for example by producing variation in oral drug absorption and thus experimental results. Digestive efficiency can vary by 8 percentage points or more between marmosets deemed outwardly healthy^14,15^, a result hypothesized to reflect the difference between animals with healthy guts and those with mild-to-moderate intestinal inflammation^15^. Prolonged subclinical CLE may result in eventual nutritional deficiencies. For example, digestive efficiency in captive marmosets was correlated with vitamin D status; animals with poor digestive efficiency were at high risk of vitamin D deficiency leading to metabolic bone disease^13,15^. A better understanding of the relationship of marmoset intestinal health to diet and nutrition will improve overall health and reduce variation in nutrient-related phenotypes.

In this observational, exploratory study, we examined digestion and energy intake in three mixed-sex groups of adult common marmosets; one at the Wisconsin National Primate Research Center (WNPRC) maintained on the WNPRC base diet (n = 28), and two at the Southwest National Primate Research Center (SNPRC), one maintained on the SNPRC base diet (n = 28) and one housed at SNPRC but originally from the New England National Primate Research Center (NEPRC) and maintained on the NEPRC base diet (n = 25). The goals of the research presented here were to characterize and assess variation in digestive efficiency in marmosets on three different base diets, to test an indirect method of assessing digestive efficiency using dietary and fecal Mn, and to characterize the differences in the feces of animals with good versus poor digestive efficiency. In addition, gross and digestible energy intake in relation to body mass, diet, and digestive abilities were assessed. This study is the most complete characterization of common marmoset food intake, digestive function, and fecal characteristics of which we are aware.

## Methods

A total of 81 adult marmosets (mixed sex, age, and body mass) housed at two primate research centers (SNPRC and WNPRC) and fed three different commercially produced base diets were included in this study. The manufacturer’s guaranteed nutrient analysis for all diets (Envigo Teklad 8794 and TD.130059, Mazuri 5M16, and Purina LabDiet AP5LK6) is provided in Supplementary Table S1. The actual macronutrient content (gross energy, fat, crude protein, neutral detergent fiber, acid detergent fiber, and ash) of the base diets was determined at the Nutritional Laboratory at the Smithsonian National Zoological Park and Conservation Biology Institute (NZP) by the assay methods described below and is provided in Table 1. All marmosets at WNPRC undergo a complete physical exam every 6 months, at SNPRC every 12 months, in addition to a physical exam including a records review prior to assignment to a research study. Based on this protocol, all the marmosets in this study were deemed healthy and eligible to be included in a research study.

**Table 1:** Composition (on a dry matter basis) of the base diets in this study based on analysis at the NZP Nutrition Laboratory. NEPRC = New England Primate Research Center, SNPRC = Southwest National Primate Research Center, WNPRC = Wisconsin National Primate Research Center.

	Envigo Teklad 8794 (NEPRC)	Envigo TD.130059 (SNPRC)	Purina LabDiet AP5LK6 (SNPRC)	Mazuri 5M16 (WNPRC)
Gross energy	4.68 kcal/g	4.23 kcal/g	4.46 kcal/g	4.52 kcal/g
Fat	6.8%	4.1%	4.8%	4.1%
Crude protein	21.9%	13.5%	21.8%	20.9%
NDF	13.7%	4.3%	7.6%	8.4%
ADF	5.2%	3.8%	2.8%	5.7%
Ash	6.0%	6.1%	6.4%	5.8%
Mn	177.6 ppm	35.6 ppm	134.5 ppm	130.2 ppm

Manufacturer identification codes, guaranteed analysis and ingredient lists for the four diets are given in Supplementary Table 1. NDF = neutral detergent fiber; ADF = acid detergent fiber.

All but one marmoset underwent two sequential 4-day digestion trials, with 3 days between trials. One animal at SNPRC included in the analysis underwent only a single 4-day trial. Based on previous studies, 4-day trials are sufficient to determine reliable measures of digestive efficiency in adult marmosets^14–16^. We conducted two consecutive trials on almost all subjects out of an abundance of caution to assess the stability of the digestive efficiency parameters measured. Several days before the initiation of the digestion trials supplemental foods were removed from the offered diet. A list of supplemental foods that were offered before the study commenced is given in Supplementary Table S1. During the digestion trials animals were fed their colony base diet only, with no supplemental foods. All food was weighed and a fresh sample frozen for later nutritional analysis. All uneaten food and feces was collected daily and frozen for later analysis. Food samples, uneaten food, and feces were dried and weighed; intake and fecal output were expressed on a dry matter basis and used to calculate the apparent digestibility of dry matter. Energy content of food and feces was determined using bomb calorimetry, enabling gross energy intake (GEI), fecal energy output, the coefficient of apparent energy digestibility (ADE), and digestible energy intake (DEI) to be calculated.

The research was approved by the Texas Biomedical Research Institute (the host institution for SNPRC) Institutional Animal Care and Use Committee under IACUC# 1519 CJ, and the University of Wisconsin, Madison College of Letters and Sciences and Office of the Vice Chancellor for Research and Graduate Education IACUC under IACUC #G005431. All protocols for this research conformed to all relevant laws, regulations, and sound research practices for research on non-human primates.

### Nutritional assays and calculations

All nutritional assays were done at the NZP Nutrition Laboratory, Washington DC. Diet samples were weighed before and after drying in a forced-air oven at 60 °C for 48 hours to determine dry matter content. Dry matter intake (DMI) was calculated by multiplying the weight of offered food by the dry matter content of the food as determined from the food samples and subtracting the dry weight of uneaten food. Gross energy (GE) of food and feces was determined via an adiabatic bomb calorimeter (6200 Isoperibolic Bomb Calorimeter and 6510 Water handling system, Parr, Moline, IL) and expressed as kcal/gram. Gross energy intake (GEI) was calculated by multiplying the energy content of the diet by the dry matter intake for each subject. Fat content of diets and feces was determined using an ANKOM fat analyzer. Nitrogen content was determined using the Dumas method (Model 2400, Perkin Elmer, Waltham, MA) and converted to percent crude protein using the conversion factor 6.25^17^. Non-soluble fiber content was determined by the Van Soest method, with neutral detergent fiber (NDF) and acid detergent fiber (ADF) determined sequentially using an ANKOM fiber apparatus. Total mineral concentration in food and feces was determined by ashing samples at 500 °C for 5 hours. The ash was solubilized in boiling nitric and perchloric acids and assayed for Mn by atomic absorption spectrometry (Model 800 Perkin Elmer Analyst Flame-Furnace Atomic Absorption Spectrophotometer, Perkin Elmer, Waltham, MA).

We assessed the utility of dietary Mn as a digestibility marker. A perfect digestibility marker is one that causes no change in palatability or digestion of the diet while being completely indigestible itself, such that 100% of the marker passes through the animal in the feces. Dietary Mn meets the first criteria, and absorption of Mn by humans, although not zero, is estimated to be low, in the range of 1–5%^18^. The total collection method for estimating digestibility is biased to over estimation because failing to collect all uneaten food will overestimate DMI, and failing to collect all feces will underestimate fecal output. Thus, we predicted that the two methods (total collection and fecal Mn) will be correlated, but that fecal Mn estimates will be lower.

From the total collection method, ADMD was calculated by 1 − dry weight of feces/DMI. Using the Mn concentrations of food and feces, ADMD was estimated using the formula:

$${\rm{ADDM}}=1\,-\,[{\rm{Mn}}\,{\rm{of}}\,{\rm{food}}]/[{\rm{Mn}}\,{\rm{of}}\,{\rm{feces}}].$$

The value for ADDM calculated from Mn concentrations and the GE of food and feces was used to calculate ADE using the formula:

$${\rm{ADE}}=1-({\rm{GE}}\,{\rm{of}}\,{\rm{feces}}/{\rm{GE}}\,{\rm{of}}\,{\rm{food}})\ast (1-{\rm{ADDM}}).$$

This formula is algebraically derived from the formulas for ADDM by total collection and ADE by total collection (ADE = (GEI − fecal GE)/GEI). The values for ADDM from Mn concentrations were used instead of the values from total collection due to several animals with poor digestion reingesting their feces, and thus thwarting total collection, and several animals with biologically unlikely high ADDM by total collection suggestion that all feces and uneaten food had not been successfully collected (see Results). Similarly, the apparent digestibility of fat was calculated from the fat content of the food and feces and the estimated ADDM from Mn by: ADFat = 1 − (%Fat of feces/%Fat of food) ∗ (1 − ADDM).

Digestible energy intake (DEI) was calculated using the calculated estimate of ADE by:

$${\rm{DEI}}={\rm{GEI}}\ast {\rm{ADE}}.$$

DEI was compared to estimated metabolic rate (MR) using the equations from Kleiber^19^, as suggested in the NRC Nutrient Requirements of Nonhuman Primates^20^:

$${\rm{MR}}=70\ast {({\rm{body}}{\rm{mass}}(\mathrm{kg}))}^{0.75}.$$

For the NEPRC and WNPRC subjects, the base diets were single items (Envigo Teklad 8794 and Mazuri 5M16, respectively, Supplementary Table S1); however, the base diet at SNPRC consists of offering animals a choice between two single item diets, a custom, purified diet (Envigo TD.130059) and Purina LabDiet AP5LK6 (Supplementary Table S1) which differed in Mn concentration (Table 1). For each SNPRC subject, intake of each diet item was calculated and the mean Mn concentration of the consumed diet was estimated by multiplying each diet’s Mn concentration by the proportion of that diet the animal consumed and then summing. A similar calculation was made to determine the GE and fat concentration of the ingested diet.

### Statistical analyses

All values are expressed as mean ± SEM. Correlation was used to test for associations between estimates of ADDM by total collection and Mn concentration, between ADE and fecal fat, and between these parameters and age and body mass. Bonferroni correction was used for multiple comparisons. Paired-sample t-test was used to examine the difference between ADDM calculated by the two methods and between the two trials. Analysis of covariance was used to test for differences in ADE and fecal fat between diets, with sex, age, and body mass as covariates for ADE, and ADE, sex, age, and body mass as covariates for fecal fat. Analysis of covariance was used to examine the association between GEI, body mass and ADE and between DEI and body mass, with diet, sex, and age as covariates. Chi-square was used to assess the differences in the proportions of high body mass animals between the diet regimes. Values were judged to be statistically different if p < 0.05, after appropriate corrections.

## Results

The average age of the marmosets in the study was 3.5 ± 0.2 years (1.7 to 10.2 years) and the mean body mass was 430.6 ± 8.7 g (315.5 to 630.5 g). On average, body mass remained constant over the trials (p > 0.1), though individual animals gained or lost weight. The means for subjects by diet are given in Table 2. Both age (p = 0.024) and body mass (p = <0.001) differed between diets. There were 38 males and 43 females in the study. The diets were generally similar in macronutrient composition (Table 1). The main difference was lower protein and Mn concentrations for the purified diet. All the diets were low fat (4.1–6.8% fat).

**Table 2:** Mean values (SEM) for subjects on each diet.

	NEPRC	SNPRC	WNPRC	P value
Age (years)	3.3 (0.2)	3.1 (0.3)	4.3 (0.5)	0.024
Body mass (g)	398.0 (11.2)	477.8 (16.9)	412.25 (10.7)	0.001
Males:Females	13:12	10:18	15:13	0.338
ADDM (%) by Mn	67.2 (1.0)	78.1 (0.8)	74.4 (0.6)	0.001
ADE (%) by Mn	69.7 (1.4)	81.3 (0.8)	75.5 (1.0)	0.001
Fecal fat (%)	7.4 (1.1)	3.7 (0.6)	10.2 (1.1)	0.021
DEI (kcal/day)	51.7 (2.2)	60.5 (2.4)	44.3 (1.3)	0.001

Statistical comparisons between diets of digestibility parameters and fecal constituents were adjusted for sex, age, and body mass. NEPRC = New England Primate Research Center diet, SNPRC = Southwest National Primate Research Center diet, WNPRC = Wisconsin National Primate Research Center diet. ADDM = apparent digestibility of dry matter; ADE = apparent digestibility of energy; DEI = digestible energy intake.

**Figure 1:**
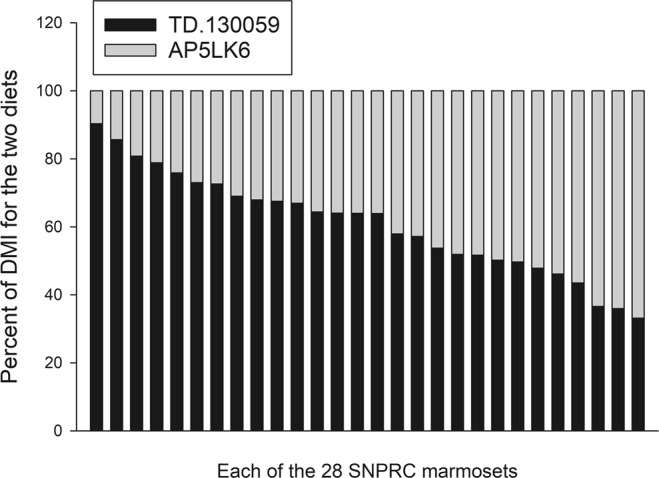
The percent of dry matter intake (DMI) from the purified diet (Envigo TD.130059) and Purina LabDiet AP5LK6 diet by individual SNPRC animals. SNPRC animals always had a choice between the two diets. Each column represents the mean proportional intake of the diets by one of the 28 SNPRC marmosets.

All of the SNPRC animals consumed both the purified and LabDiet diets (Fig. 1), with the purified diet being preferred, on average (mean of 66.8 ± 2.8% of DMI; 33.2–90.3%). The calculated Mn concentration of the consumed SNPRC diet ranged from 45.0 ppm to 101.6 ppm.

**Figure 2:**
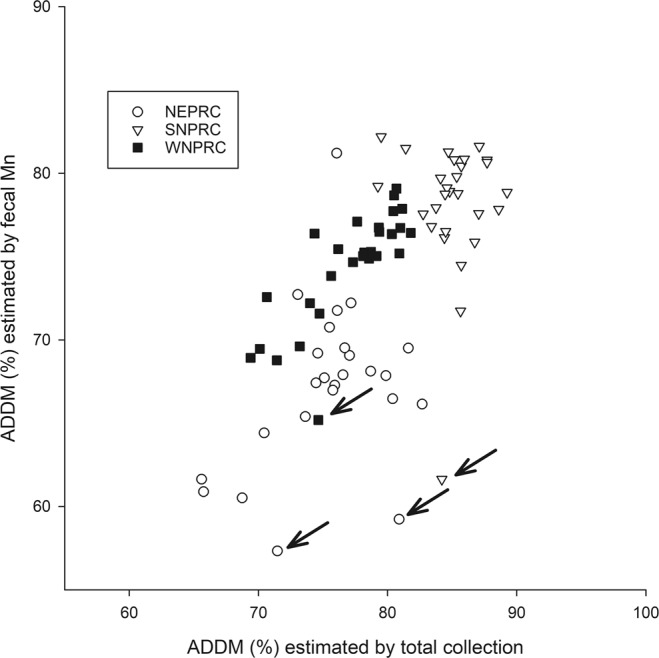
The relationship between apparent dry matter digestibility (ADDM) estimated by the total collection method and by fecal Mn concentration (r = 0.658, p < 0.001). Two animals on the NEPRC diet, one on the SNPRC diet, and one on the WNPRC diet had estimates of ADDM by fecal Mn that were more divergent from their estimate by total collection than the other values (arrows).

Estimates for ADDM from the total collection method and by fecal Mn concentration were positively correlated (r = 0.658, p < 0.001; Fig. 2). Mean ADDM by Mn was lower than ADDM estimated by total collection for all diets (mean difference = 5.8%, p < 0.001). There were two conspicuous outliers on the NEPRC diet, one on the SNPRC diet and one on the WNPRC diet (Fig. 2) where the estimate by total collection was far greater than ADDM estimated by fecal Mn, as well as several individuals on the SNPRC diet with ADDM by total collection of almost 90%. We hypothesize these anomalous data points represent a failure to completely collect feces and uneaten food, in part due to reingestion of feces by some subjects.

**Figure 3:**
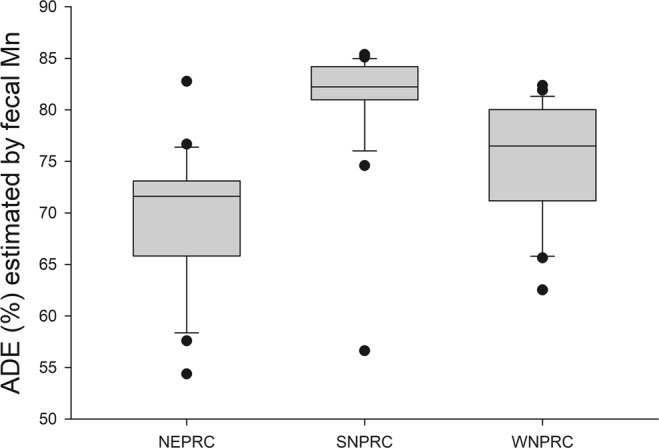
Box plots of the apparent digestibility of energy (ADE) by diet. The line within represents the median, the box comprises the 25^th^ percentile to the 75^th^ percentile. The whiskers (error bars) go from the 10^th^ percentile to the 90^th^ percentile. Points outside the whiskers indicate the outliers.

**Figure 4:**
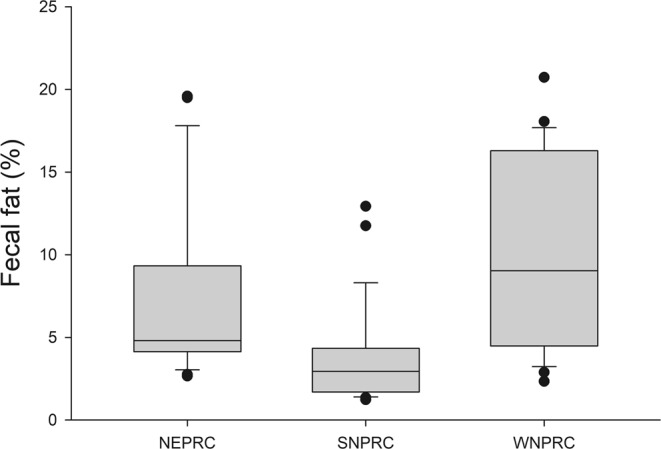
Box plots of the percentage of fecal fat by diet. The line within represents the median, the box comprises the 25^th^ percentile to the 75^th^ percentile. The whiskers (error bars) go from the 10^th^ percentile to the 90^th^ percentile. Points outside the whiskers indicate the outliers.

There was a significant effect of diet on ADDM, ADE, and fecal fat, after accounting for sex, age and body mass, with the SNPRC diet producing the highest values for digestibility, the lowest values for fecal fat, and generally the lowest variation between animals (Table 2; Figs 3 and 4). There was no significant difference between males and females in any of these parameters, and no interaction between sex and diet. There were significant effects of body mass on ADDM, ADE, and fecal fat. Body mass was positively correlated with both digestibility parameters (r = 0.472 and 0.496 for ADDM by Mn and ADE, respectively; p < 0.001). Fecal fat was positively correlated with age (r = 0.438, p = 0.001) and negatively correlated with body mass (r = −0.433; p = 0.001). Age and body mass were not associated (r = −0.008); p = 1.0).

**Figure 5:**
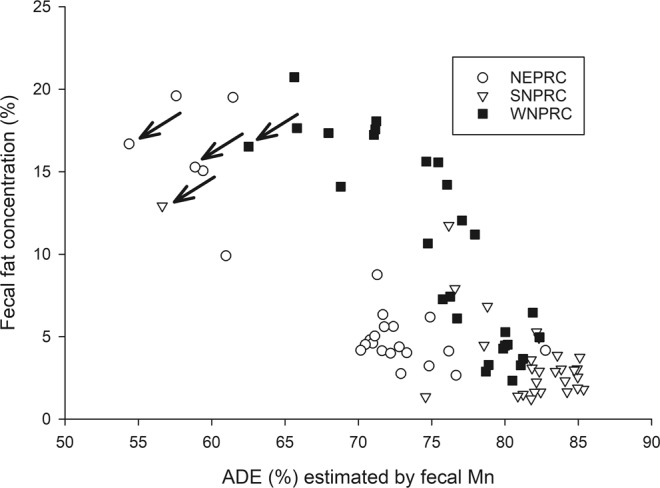
The relationship between apparent digestibility of energy (ADE) and fecal fat concentration for all animals. The four animals that had anomalously high apparent digestibility of dry matter (ADDM) by the total collection method compared to their estimate of ADDM by fecal Mn all had high fecal fat (arrows).

The NEPRC diet had the lowest values for ADE, but the WNPRC diet produced the greatest proportion of animals with high fecal fat (Table 2; Fig. 4). Half of the WNPRC animals had fecal fat above 10% compared to 20% of the NEPRC animals and only 7% of the SNPRC animals. Using analysis of covariance, both ADE and diet are significant factors explaining fecal fat concentration (R^2^ = 0.792, p < 0.001 for both). Fecal fat was strongly, negatively correlated with ADE (r = −0.739; p < 0.001; Fig. 5). Note that the four animals with anomalous high ADDM by total collection (two NEPRC and one each for SNPRC and WNPRC) had high fecal fat concentrations (Fig. 5), consistent with their low ADDM by fecal Mn.

The estimated values for ADFat ranged from strongly negative (−47%) to 94%. The mean estimated ADFat for animals with more than 10% fat in their feces was essentially zero (2.5 ± 5.7%). For animals with less than 5% fat in their feces ADFat was normally distributed with a mean of 85.1 ± 0.9% and a median of 85.7%. The proportion of animals with fat below 5% differed between diets, with 22 of 28 SNPRC animals, 13 of 25 for NEPRC, and only 8 of 28 WNPRC subjects having fecal fat below 5%.

Based on that result, we examined ADE for all marmosets with fecal fat under 5% to assess the differences in digestibility due to diet, under the assumption that those marmosets were likely to have “healthy” intestinal tracts. The diets significantly differed in ADE (p < 0.001); SNPRC diet had the highest ADE (84.8 ± 0.4%), followed by the WNPRC diet (79.3 ± 0.3%) and the NEPRC diet (72.9 ± 0.7%).

**Figure 6:**
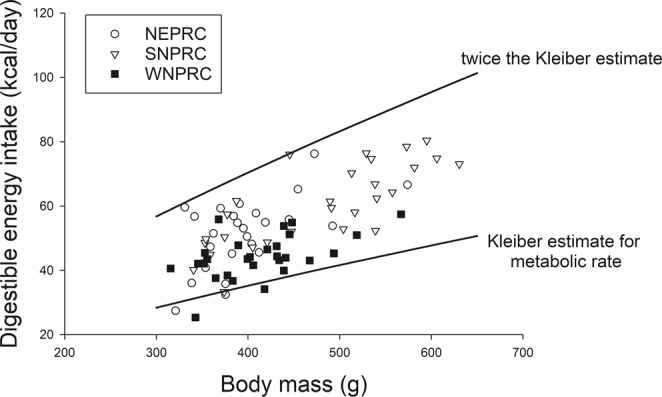
The relationship between digestible energy intake and body mass for all animals. The Kleiber estimate for daily metabolic rate (MR) is: MR = 70 * (body mass in kg)^0.75^. The two trend lines give the Kleiber MR estimate and the value for twice the Kleiber MR estimate.

**Figure 7:**
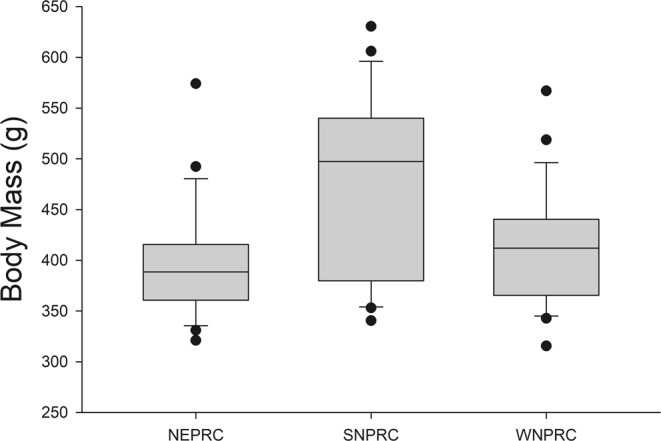
Box plots of body mass by diet. The line within represents the median, the box comprises the 25^th^ percentile to the 75^th^ percentile. The whiskers (error bars) go from the 10^th^ percentile to the 90^th^ percentile. Points outside the whiskers indicate the outliers.

Body mass and DEI were positively correlated (r = 0.700, p < 0.001; Fig. 6). The ANCOVA results indicated that age and sex were not significant factors for GEI or DEI. Diet and body mass were significant factors explaining GEI (p < 0.001 for both), while ADE tended to be associated (p = 0.065). After adjustment for body mass and ADE, GEI was not different between NEPRC and SNPRC (p = 0.399), while NEPRC and SNPRC GEI was greater than for WNPRC marmosets (p < 0.001 and 0.012, respectively). For DEI, diet and body mass were the only significant factors (p < 0.001 for both). After adjusting for body mass, DEI for animals on the NEPRC and SNPRC diets did not differ, but both were greater than DEI for WNPRC marmosets (p = 0.001 and 0.003, respectively). SNPRC animals had the numerically highest mean DEI and also the highest mean body mass (Fig. 7), with the highest proportion of very high body mass animals (above 450 g). More than half (57%) of SNPRC animals were above 450 g compared to 16% and 14% for NEPRC and WNPRC, respectively (Chi-square = 15.554, df = 2, p < 0.001), and 50% of the SNPRC subjects were above 500 g, compared to 4% and 7% for NEPRC and WNPRC, respectively (Chi-square = 21.799, df = 2, p < 0.001.

Because selection bias could have resulted in an oversampling of high body mass animals on the SNPRC diet we examined colony records for the time period of the study. Based on colony records, the mean body masses of all adult, non-pregnant animals in the WNPRC (438 ± 5 g), and SNPRC (456 ± 7 g) colonies, and the animals maintained on the NEPRC diet but housed at SNPRC (441 ± 7 g) were not significantly different, though the difference between SNPRC and WNPRC approached significance (p = 0.065). The SNPRC colony had a significantly greater proportion of animals above 500 g (36.1% versus 22.1% and 18.9% for NEPRC and WNPRC marmosets; Chi-square = 9.829, df = 2, p = 0.007). The median body weight for SNPRC (480 g) was above the mean, suggesting a skew towards higher body weight. The median body weight for both WNPRC and NEPRC colonies was 440 g, essentially equal to the mean values.

## Discussion

Our findings confirm that apparently healthy marmosets can vary widely in digestive ability when fed the same diet. We hypothesize the variation derives from varying levels of intestinal inflammation, however, our study did not directly address the causes of the variability in digestive efficiency. A previous study found an association between low digestive efficiency and vitamin D deficiency that was proposed to be related to lipid malabsorption^15^. In this study, animals with low digestive efficiency had higher fecal fat concentrations (Fig. 5), consistent with digestive difficulties leading to lipid malabsorption, lending support to the hypothesis that vitamin D-related issues in marmosets may be caused by intestinal inflammation or other causes of digestive difficulties. Animals with more than 10% fat in their feces appeared to essentially have no fat absorption. Half of the WNPRC animals had fecal fat above 10%, suggesting this group would be at high risk for vitamin D deficiency; however, the WNPRC diet has substantial added vitamin D that may serve to mask malabsorption difficulties, as frank signs of vitamin D deficiency were not seen.

Our results indicate that dietary Mn can be used as a digestibility marker to estimate digestive efficiency in marmosets fed diets that have consistent levels on Mn. Marmosets, like humans, appear to absorb only a small fraction of dietary Mn. Fecal Mn concentration will underestimate apparent digestibility, since the concentration of Mn in the feces will be lower than if it was completely indigestible. Because there will be some variation in the percentage of Mn absorbed between individuals, this technique would not be able to distinguish between animals that differed by only a few percentage points in ADDM, but our data show that it can distinguish between healthy animals and poor digesters. We caution that this result may not extend to other mammals, though we deem it likely that dietary Mn will be poorly absorbed by most primates, with the possible exception of members of the Colobinae, due to their foregut fermentation system.

The concentration of fat in the feces may serve as a useful diagnostic tool to identify animals at risk of digestive pathology and related health problems. Based on our data, we suggest that a fecal fat concentration under 5% is indicative of a healthy marmoset. A fecal fat concentration above 10% is indicative of an animal that may be facing significant health issues. Simpler methods to assess the fat in marmoset feces should be explored to use as a management tool.

In addition to the health consequences, these results have implications for research studies. All the animals in this study were outwardly healthy and may have been chosen for a research study. It is likely, however, that the animals with low digestibility and fecal fat over 5% differed in nutritional health from animals with fecal fat under 5%, especially for animals with fecal fat above 10%. If chosen for a study, these animals may generate unaccounted for variation in the results due to their different metabolic/nutritional states. Concern regarding variability in digestive efficiency would be especially true for research such as oral drug studies. We suggest screening and selecting animals with less than 5% fecal fat would lessen variability of research results within and between colonies.

The study results demonstrated that there is extensive variation among individuals in digestive efficiency for all three diets. All diets had poor digesters with high fecal fat. The results from marmosets with fecal fat under 5% indicate that the three diets are inherently different in their digestibility by marmosets, but no value judgement can be placed on whether a diet is better or worse based on that criteria. Where a value judgement might be appropriate is the extent of variability of diet digestibility between marmosets. Whether or not diet contributes to the genesis of intestinal inflammation in marmosets cannot be determined from these data, but high variability in digestive efficiency with a large proportion of poor digesters suggests that a diet is challenging to individuals susceptible to intestinal inflammation. It is possible that a diet such as the purified diet used at SNPRC may be less challenging to those animals, and feeding animals with poor digestive function easily digested diets may be of benefit to them.

The diet used at SNPRC had the highest apparent digestibility, lowest variation among individuals (Fig. 3), and fewest animals with high fecal fat (Fig. 4). However, SNPRC colony animals were also the heaviest, with a majority over 450 g and over one-third above 500 g. Previous studies have shown that marmosets above 435 g have poor metabolic profiles indicating obesity^7,8^. Although the SNPRC diet appeared to be associated with more consistent digestive performance, possibly related to better intestinal health or simply by being less of a challenge to poor digesters, it also may be obesogenic. A palatable, highly digestible diet, such as the SNPRC diet, may not be the best choice for overall colony health under ad lib feeding regimes, as it could encourage overeating leading to excessive weight gain. Lower digestibility diets may be of benefit in combating obesity in captive marmosets by reducing caloric intake while maintaining normal levels of food intake.

The food intake data are consistent with basic principles. Larger animals tended to ingest a greater amount of calories, though there was substantial variation at any body mass. Some of the variation in GEI is explained by ADE, as animals with low digestive efficiency increased GEI. Because weights were generally stable over the study, it appears that even the poor digesters were able to regulate energy intake sufficiently to maintain body mass homeostasis. Whether they were able to maintain sufficiency for fat soluble vitamins such as vitamins A and D is not known and would be a concern, given the high fecal fat concentrations for these animals. Previous studies suggest this would not be the case (Jarcho *et al*.)^15^.

Even after accounting for differences in digestive efficiency by calculating DEI there was significant variation among animals with similar body masses (Fig. 6). The recommendations from the National Research Council (NRC, 2003)^20^ suggest that a reasonable estimate of energy requirement is twice the estimated metabolic rate, using Kleiber’s equation (70 * (body mass in kg)^0.75^) for metabolic rate^19^. DEI for the animals in this study ranged from approximately estimated metabolic rate to almost two times the Kleiber estimation (Fig. 6). A value of 1.5 times the Kleiber estimate may be a better target for captive marmosets. Although callitrichid monkeys, such as marmosets, have a resting metabolic rate similar to the Kleiber estimate during waking hours, during night time sleep these monkeys drop both body temperature and metabolic rate significantly. Sleeping metabolic rate is approximately two-thirds of waking resting metabolic rate^16,21–24^. If we assume common marmosets on average expend energy at about twice the Kleiber metabolic rate estimate during 12 hours of awake time and at about 70% of that estimate during sleep, total energy expenditure would be approximately 1.35 times the Kleiber estimate for metabolic rate, consistent with our data. Both theory and data suggest that a better estimate of marmoset energy requirement would be 1.35 - 1.5 times the Kleiber metabolic rate estimate.

In summary, our study confirmed previous findings that captive marmosets can vary widely in digestive efficiency when fed identical diets. Previous research had shown an association between low digestive efficiency and low vitamin D status^15^. Our study has demonstrated an association between low digestive efficiency and high fecal fat, suggesting lipid malabsorption and consistent with a risk for fat soluble vitamin deficiency. Our data suggest that a fecal fat content of less than 5% is indicative of good intestinal health in marmosets. Based on our results we suggest that there may be value to feeding marmosets with poor digestive function a highly digestible diet, such as the purified diet used at SNPRC. However, for healthy animals, our data suggest that a highly digestible diet increases the risk of obesity. Our data further suggests that the current NRC energy requirements for non-human primates^20^ overestimates the actual energy requirement for captive marmosets. We propose that a better energy requirement estimate for captive marmosets to be 65–75% of the NRC suggested value.

## Supplementary information

Manufacturer’s guaranteed analysis and ingredient list

## Data Availability

The datasets generated during and/or analysed during the current study are available from the corresponding author on reasonable request.
