# Supplementary material for: Diet, digestion and energy intake in captive common marmosets (Callithrix jacchus): research and management implications
Source: Sci Rep. 2019 Aug 20;9:12134. doi: 10.1038/s41598-019-48643-x (PMC6702194; doi:10.1038/s41598-019-48643-x)
Supplement: Supplementary file 1 — Manufacturer’s guaranteed analysis and ingredient list [file 41598_2019_48643_MOESM1_ESM.docx]

Diet, digestion and energy intake in captive common marmosets (*Callithrix jacchus*): research and management implications.

Michael L. Power^1^, Jessica Adams^2^, Kirsten Solonika^3^, Ricki J. Colman^3,4^, Corinna Ross^2,5^, Suzette D. Tardif^2^

^1^ Conservation Ecology Center, Smithsonian Conservation Biology Institute, Washington DC, USA

^2^ Southwest National Primate Research Center, Texas Biomedical Research Institute, San Antonio TX, USA

^3^ Wisconsin National Primate Research Center, Madison WI, USA

^4^ Dept. of Cell & Regenerative Biology, School of Medicine and Public Health, University of Wisconsin, Madison, WI, USA

^5^ Texas A&M University San Antonio, San Antonio, TX, USA

Supplemental Table S1. Manufacturer’s guaranteed analysis and ingredient list for the four base diets, and the list of supplemental foods typically offered to the animals before the study began. The supplemental foods were discontinued several days before the digestion trials started.

|  | Envigo Teklad 8794  (NEPRC) | Envigo TD.130059 (SNPRC) | Purina LabDiet AP5LK6 (SNPRC) | Mazuri 5M16 (WNPRC) |
| --- | --- | --- | --- | --- |
| Crude protein | 20.0% | 14.4% | 21.3% | 19.0% |
| Fat | 10.0% | 5.2% | 7.8% | 6.0% |
| Neutral detergent fiber | 12.6% | 5.0% | 6.0% | 9.9% |
| Ash | 6.2% |  | 4.6% | 4.7% |
| Ingredients listed in descending order by amount  , | | |  |  |
| Envigo Teklad 8794 | Ground corn, ground wheat, wheat middlings, wheat germ, dehulled soybean meal, corn gluten meal, sucrose, soybean oil, dried beet pulp, porcine fat, egg product, dried whey, calcium carbonate, dehydrated alfalfa meal, fish meal, casein, mineral and vitamin supplements | | | |
| Envigo TD.130059 | Dextrin, sucrose, casein, mineral mix, soybean oil, cellulose, sodium chloride, vitamin mix, L-Cystine, choline bitartrate, vitamin C, vitamin E, vitamin K (phylloquinine), TBHQ (antioxidant) | | | |
| Purina LabDiet AP5LK6 | Glucose, dehulled soybean meal, ground corn, casein, wheat middlings, gelatin, ground wheat, soybean oil, dried egg product, fructose, wheat germ, corn gluten meal, dried whey, calcium carbonate, dried beet pulp, corn oil, brewers dried yeast, dehydrated alflfa meal, wheat bran, citric acid (preservative), cicalcium phosphate, berry flavor, flaxseed oil, sodium hexametaphosphate, xanthan gum, mineral and vitamin supplements | | | |
| Mazuri 5M16 | Glucose, dehulled soybean meal, ground corn, casein, gelatin, ground beet pulp, corn gluten, wheat middlings, fructose, ground aspen, sucrose, calcium carbonate, soybean oil, ground wheat, powdered cellulose, corn oil, apple pomace, dried egg product, sodium hexametaphosphate, wheat germ, dried whey, citric acid, dicalcium phosphate, berry flavor, brewers dried yeast, flaxseed oil, dehydrated alfalfa meal, wheat bran, xanthan gum, ground flax seeds, mineral and vitamin supplements | | | |
| Supplemental foods typically offered to the marmosets; only a few items are offered each day | Hard-boiled egg, garbonzo beans, cottage cheese, apple, banana, orange, dried papaya, raisins, dried cranberries, celery, carrot, lettuce, green beans, frozen peas | | | |
